# Long read sequencing on its way to the routine diagnostics of genetic diseases

**DOI:** 10.3389/fgene.2024.1374860

**Published:** 2024-03-06

**Authors:** Giulia Olivucci, Emanuela Iovino, Giovanni Innella, Daniela Turchetti, Tommaso Pippucci, Pamela Magini

**Affiliations:** ^1^ IRCCS Azienda Ospedaliero-Universitaria di Bologna, Bologna, Italy; ^2^ Department of Surgical and Oncological Sciences, University of Palermo, Palermo, Italy; ^3^ Department of Medical and Surgical Sciences (DIMEC), University of Bologna, Bologna, Italy; ^4^ Medical Genetics Unit, IRCCS Azienda Ospedaliero-Universitaria di Bologna, Bologna, Italy

**Keywords:** long read sequencing, molecular diagnosis, genetic diseases, structural variants, tandem repeats, single nucleotide variants, methylation, RNA sequencing

## Abstract

The clinical application of technological progress in the identification of DNA alterations has always led to improvements of diagnostic yields in genetic medicine. At chromosome side, from cytogenetic techniques evaluating number and gross structural defects to genomic microarrays detecting cryptic copy number variants, and at molecular level, from Sanger method studying the nucleotide sequence of single genes to the high-throughput next-generation sequencing (NGS) technologies, resolution and sensitivity progressively increased expanding considerably the range of detectable DNA anomalies and alongside of Mendelian disorders with known genetic causes. However, particular genomic regions (i.e., repetitive and GC-rich sequences) are inefficiently analyzed by standard genetic tests, still relying on laborious, time-consuming and low-sensitive approaches (i.e., southern-blot for repeat expansion or long-PCR for genes with highly homologous pseudogenes), accounting for at least part of the patients with undiagnosed genetic disorders. Third generation sequencing, generating long reads with improved mappability, is more suitable for the detection of structural alterations and defects in hardly accessible genomic regions. Although recently implemented and not yet clinically available, long read sequencing (LRS) technologies have already shown their potential in genetic medicine research that might greatly impact on diagnostic yield and reporting times, through their translation to clinical settings. The main investigated LRS application concerns the identification of structural variants and repeat expansions, probably because techniques for their detection have not evolved as rapidly as those dedicated to single nucleotide variants (SNV) identification: gold standard analyses are karyotyping and microarrays for balanced and unbalanced chromosome rearrangements, respectively, and southern blot and repeat-primed PCR for the amplification and sizing of expanded alleles, impaired by limited resolution and sensitivity that have not been significantly improved by the advent of NGS. Nevertheless, more recently, with the increased accuracy provided by the latest product releases, LRS has been tested also for SNV detection, especially in genes with highly homologous pseudogenes and for haplotype reconstruction to assess the parental origin of alleles with *de novo* pathogenic variants. We provide a review of relevant recent scientific papers exploring LRS potential in the diagnosis of genetic diseases and its potential future applications in routine genetic testing.

## 1 Introduction

Molecular diagnosis has an important impact on clinical management of patients with genetic diseases and their families. It confers the certification of a chronic and often disabling condition with dedicated healthcare pathways, specific treatment options and, still in few cases, personalized therapies. The causative DNA alteration can be searched in future pregnancies, allowing informed reproductive choices, and, especially in the case of hereditary tumor predisposition syndromes, in healthy family members, who can benefit from the most appropriate clinical management based on the resulting risk. Reporting times are obviously important to ensure rapid communication and timely interventions, both in the postnatal and even more in the prenatal settings.

Genetic testing started in 1960 with karyotyping and later evolved rapidly with the introduction of molecular analysis using DNA as probe (i.e., fluorescence *in situ* hybridization) or target (i.e., Sanger sequencing). This first advancement from the study of all chromosomes to specific genomic regions brought a gain in resolution at the expense of the investigation scale, making the diagnosis dependent from target selection guided by clinical hypotheses. The next technological advancement was aimed at achieving high resolution in the context of genome-wide analysis, allowing clinically unbiased testing. The first techniques with these features were microarrays, that brought a huge revolution in the cytogenetic field, replacing karyotype analysis as first-tier test for neurodevelopmental disorders (NDDs) and congenital malformations with an increase in diagnostic yield from 3% to 15%–20% (Miller et al., 2010). The advent of second or next-generation sequencing technologies based on short reads marked the greatest progress in genetic medicine by the analysis of the entire exome or genome at nucleotide-level resolution, finding pathogenic variants in nearly 30%–38% of NDD cases or even higher percentages of patients with specific genetic conditions (Nurchis et al., 2023; van der Sanden et al., 2023). This technological evolution impacted not only on detection rates and diagnostic yields but also on laboratory routine. Faster and simpler wet lab protocols increased the number of patients that could be analyzed simultaneously and reduced turnaround times. However, all these techniques are far from being completely efficient, even if combined and applied sequentially, missing the molecular diagnosis in about half of the patients with genetic conditions (Haghighi et al., 2018; Boycott et al., 2019). Indeed, apart from interpretative and clinical issues, including the partial knowledge of the functions of genes and genomic regions or the incomplete phenotypic characterization of patients preventing accurate genotype-phenotype correlations, technical limitations contribute substantially to missing diagnoses (Mastrorosa et al., 2023). In particular, repetitive, highly homologous and GC-rich sequences are difficult to analyze with current genetic tests, and strategies specifically developed to reach these hard genomic regions are still laborious, time-consuming and low-sensitive (i.e., southern-blot for repeat expansion or long-PCR for genes with highly homologous pseudogenes) (Mandelker et al., 2016). In addition, the detection of structural variants (SVs) through short read sequencing (SRS) is still limited and requires advanced bioinformatic tools that have not yet been incorporated into the routine pipelines (Neerman et al., 2019).

The ability of third generation sequencing or long read sequencing (LRS) technologies to sequence long molecules of nucleic acids with improved mappability overcomes the technical limitations of SRS, potentially impacting on detection rate and diagnostic yield (Mastrorosa et al., 2023).

SRS technologies, particularly those based on sequencing-by-synthesis, have shown exceptional accuracy, reaching 99.9% base-call accuracy (Lin et al., 2021). However, reads are usually a few hundred base pairs long, limiting the ability to map reads, especially in low-complexity repetitive loci and duplicated regions, and to decipher structural variants or tandem repeats. LRS, on the other hand, enables sequencing of DNA fragments longer than 10,000 bp. At present, the two major LRS technologies, namely Pacific Biosciences and Oxford Nanopore Technology, are based on different approaches. The first is based on single molecule real-time sequencing and allows real-time detection of nucleotide incorporation events using a DNA polymerase, with high accuracy (up to 99.8% with circular consensus sequencing) (Wenger et al., 2019). One limitation of this technology is, however, the high cost of equipment and supplies. Nanopore-based sequencing detects changes in the ionic current during translocation of a single-stranded molecule of DNA/RNA through a biosensor (nanopore) to generate even longer reads (10–100 kb with standard long-reads, >100 kb with ultra-long reads, up to a described maximum length of about 2 Mb) (Payne et al., 2019), enabling access to long repetitive regions. This technology has been characterized by lower accuracy and particular high error rate in single nucleotide variant (SNV) and indel calling, which, however, is improving due to advances in chemistry and algorithms (Ni et al., 2023). The intent of this review is not to make a comparison between the two existing LRS technologies. For this reason, we do not make any further distinction between these different technological approaches, referring to them collectively as LRS. Descriptions of both technologies, their comparison and data analysis tools are reported in several scientific publications. We provide some of them to address readers for further information (Amarasinghe et al., 2020; Logsdon et al., 2020; Amarasinghe et al., 2021; Athanasopoulou et al., 2022; Mastrorosa et al., 2023; van Dijk et al., 2023).

Human reference genome releases commonly used to align sequences (GRCh37 and GRCh38) are highly fragmented and contain unresolved portions. The advent of LRS technologies allowed the release of whole genome haplotype resolved assemblies (Nurk et al., 2020; Cheng et al., 2021), including pericentromeric and subtelomeric regions and the short arms of acrocentric chromosomes (Nurk et al., 2022), and the generation of benchmarks for variants in hard-to-assess disease-genes (Wagner et al., 2022), improving sequencing and pathogenic alteration identification.

Advantages of LRS clinical application might concern not only the enhanced detection of missing variants but also reduced turnaround times, from blood sampling to analysis results and report, being based on rapid wet lab protocols for sample processing. Moreover, LRS potentially allows the evaluation of different alteration types in the same analysis (i.e., methylation, SNVs and SVs), avoiding the sequential application of inconclusive tests and therefore bringing a cost advantage.

We provide here a review of the recent scientific literature exploring LRS potential applied to the identification of disease-related genetic and epigenetic alterations, focusing on diagnostic and clinical implications and highlighting the advantages over current genetic testing.

## 2 Structural variants

SVs are a large class of genomic variations that differ greatly in type and size. They usually range from 50 bp to well over megabases and include both balanced and unbalanced alterations. Balanced SVs are changes in DNA structure, such as inversions of a genetic fragment or translocations of genomic segments within or between chromosomes. Unbalanced SVs, also referred to as copy number variations (CNVs), include gains and deletions of genetic material. Due to their remarkable size, SVs are considered the largest source of genetic variation in terms of the number of nucleotides involved and they are implicated in several Mendelian disorders through different mechanisms, disrupting either regulatory or coding sequences or altering the copy number of dosage-sensitive genes (Carvalho and Lupski, 2016; Middelkamp et al., 2019; Ho et al., 2020). However, compared to other classes of variants such as SNVs, they are still largely understudied due to the technical limitations of identification methods (Amarasinghe et al., 2020; Bolognini and Magi, 2021).

### 2.1 Current diagnostic analyses for SV detection

Various techniques were developed over the last 60 years to allow a genome-wide identification of different types of SVs in clinical diagnosis. These range from cytogenetic analyses (i.e., karyotyping), oligonucleotide-based technologies (i.e., Array Comparative Genomic Hybridization [aCGH] and Single Nucleotide Polymorphism [SNP] array) to short-reads whole genome sequencing (WGS) (Liu et al., 2022a). However, each of these techniques has several weaknesses, which are summarized in Table 1.

**TABLE 1:** Comparison of current diagnostic techniques for the detection of structural variants. CNV, copy number variant; MLPA, multiplex ligation-dependent probe amplification; SRS, short read sequencing; SV, structural variant.

	Karyotyping	Microarrays	MLPA	SRS technologies
Chromosomal alterations detected	Polyploidies and aneuploidies Balanced SVs CNVs	Aneuploidies CNVs	CNVs	Balanced SVs CNVs
Resolution	>5–10 Mb	Variable according to array design, generally ≥10 kb	Exon level	All range of sizes from 50 bp
Analysis scale	Whole genome	Whole genome	Target regions	Whole genome
Limitations	Low resolution Time-consuming and labor-intensive	Inability to detect balanced SVs Low sensitivity for mosaic CNVs Inability to map the insertion position of duplicated regions	Targeted analysis Low sensitivity for mosaic CNVs	Low accuracy and reproducibility Inability to identify SVs in repeated regions or complex genomic regions

Karyotyping provides a complete overview of the human genome and enables the detection of large, clinically significant chromosomal rearrangements. It can detect balanced and unbalanced SVs with a limited resolution of 5–10 Mb, preventing the identification of smaller SVs. Array-based approaches or microarrays are extensively used in clinical genetics: with a diagnostic yield of 15%–20%, they represented the first-tier genetic test for NDD (Miller et al., 2010) for many years, until the clinical application of SRS pushed molecular diagnosis to at least 31% of NDD patients (Srivastava et al., 2019). Although they provide higher resolution than karyotyping, diagnostic microarrays generally detect CNVs larger than 10–50 kb, with low resolution for mosaic alterations, and miss all balanced or smaller SVs. The identification of SVs, together with SNVs, from SRS data would be the ideal solution to save time and money. Continuous advances in short-read technologies based on paired-end sequencing have led to an unprecedented development of algorithms and tools that can potentially detect all types of SVs with breakpoint resolution. However, despite the more than 80 bioinformatic tools that have been developed for the detection of SVs in short-read WGS data, several benchmark studies have shown that none of these tools has high sensitivity and accuracy for all SV types and sizes (Cameron et al., 2019; Kosugi et al., 2019). The identification of SVs spanning several hundred to thousands of base pairs with short reads (150–250 bp) represents the greatest SRS challenge. Reads are typically smaller than SVs, making the signal reconstruction difficult to model with available methods. In addition, studies also showed a high number of false positives and the inability to detect SVs in low complexity regions consisting of tandem or simple repeat sequences or in genomic loci characterized by segmental duplications, which are hotspots for SVs (Alkan et al., 2011; Mills et al., 2011; Zook et al., 2020). These problems strongly limit the application of SRS to the identification of SVs in diagnostics, where microarrays remain the method of choice for the detection of CNVs and karyotyping is still the only reliable technique for balanced SVs, despite its low resolution.

### 2.2 Clinical application of LRS for SV identification

LRS has been proposed as the sequencing alternative to overcome the limitations of existing technologies and increase the accuracy of SV detection (Branton et al., 2008; Eid et al., 2009). The reads generated by LRS can completely span SVs, generate highly reliable alignments at breakpoints, resolve complex structural variants (cxSVs) involving three or more breakpoint junctions and directly reconstruct haplotypes (Sedlazeck et al., 2018; Hiatt et al., 2021). Since the read length is usually longer than the most common repeated sequences, LRS technology significantly increases the sensitivity in detecting SVs and, according to a study by Ebert et al. can identify more than 68% of SVs that are undetectable with SRS (Sedlazeck et al., 2018; Ebert et al., 2021; Olson et al., 2022). Over the past few years, several studies have shown the potential of LRS in improving the diagnostic yield in Mendelian diseases, especially in cases that remain unsolved after SRS and may be carriers of SVs or complex events that are difficult to detect with current technologies because breakpoints are located in repetitive regions. In one of the first clinical applications of LRS Merker et al. identified a causative deletion of 2.2 kb in the *PRKAR1* gene in a patient with Carney complex (Merker et al., 2018). In the same year, Miao et al., 2018 used LRS to detect a deletion of 7.1 kb in the *G6PC* gene missed by SRS and affecting the non-mutated allele, confirming the clinically suspected diagnosis of recessive glycogen storage disease type Ia syndrome. Another example of LRS utility in undiagnosed recessive disorders was provided by Sano et al., 2022, who studied a cohort of 15 patients with retinitis pigmentosa and carriers of pathogenic variants in one allele of the *EYS* gene: whole-genome LRS was able to detect all the causative SNVs previously identified and two large deletions (376 kb and 395 kb) encompassing the coding sequence of *EYS* in two patients. In a patient with Usher syndrome and a maternally inherited *PCDH15* exonic deletion identified by CNV analysis on SRS data, LRS allowed a better characterization of the intragenic loss and the identification of a 4.6 Mb inversion on the paternal allele, generating two aberrant fusion transcripts, PCDH15-LINC00844 and BICC1-PCDH15 (Vaché et al., 2020).

Additional studies further confirmed the utility of LRS as an effective technology for the identification of clinically relevant SVs missed by standard genetic tests (Mizuguchi et al., 2019; Hiatt et al., 2021; Miller et al., 2021; Lecoquierre et al., 2023; Ohori et al., 2023). Damián et al., 2023 effectively characterized two cases of congenital aniridia after a decades-long diagnostic odyssey. In one of these cases, they found a cryptic 4.9 Mb *de novo* inversion disrupting intron 7 of *PAX6* while in the other they were able to reclassify a balanced t (6; 11) translocation cytogenetically identified years earlier and erroneously considered as non-pathogenic, by precisely mapping its breakpoint within a *PAX6* enhancer.

The precise definition of SV breakpoints is one of the most important clinical applications of LRS. Examples include the accurate assessment of the genomic position with nucleotide-level resolution of a deletion in the *BBS9* gene in a patient with Bardet-Biedl syndrome (Reiner et al., 2018) and of a balanced reciprocal t (X; 20) translocation, disrupting the X-linked *ARHGEF9* gene, associated with developmental and epileptic encephalopathy, that was previously detected but not accurately mapped by karyotype analysis in a girl with intellectual disability and seizures (Dutta et al., 2019). In a family with three non-contiguous *DMD* duplications identified through standard testing, the precise breakpoint definition by LRS, showing the presence of an intact copy of the gene, contributed to the reclassification of the rearrangement as benign, with important implications for genetic counseling, especially in prenatal setting (He et al., 2022). Mariya et al., 2023 used a targeted LRS approach to sequence the breakpoint junctions of a *PLP1* duplication and a *MECP2* duplication/triplication causing Pelizaeus‐Merzbacher disease and *MECP2* duplication syndrome, respectively. In the latter case, the authors were able to map one of the *MECP2* copies 45 Mb apart from the original position, avoiding a possible misinterpretation of the pathogenic allele by short tandem repeat haplotyping in pre‐implantation genetic testing, in case of recombination events occurring within this 45 Mb interval. The inability of mapping the extra copy or copies of genomic gains is one of the main limitations of current diagnostic analyses widely applied to genetic conditions (i.e., microarrays and Multiplex Ligation-dependent Probe Amplification [MLPA]), impairing the correct clinical classification of these CNVs.

The impact of this clinical application of LRS has been studied also for cancer susceptibility genes (CSGs), in which CNVs are usually investigated by MLPA after inconclusive SRS results, but other types of SVs are not routinely evaluated. Thibodeau et al. evaluated the utility of LRS in resolving candidate germline SVs in CSGs detected through short-read genome sequencing in a cohort of 669 advanced cancer patients. They were able to confirm eight simple pathogenic or likely pathogenic SVs, resolve three additional variants whose impact could not be fully elucidated through SRS, and classify a recurrent alteration on chromosome 16p13 as a sequencing artifact and one complex rearrangement on chromosome 5q35 as likely benign, obviating the need for further clinical assessment (Thibodeau et al., 2020). These results demonstrated that LRS can improve the validation, resolution, and classification of germline SVs in CSGs. A similar approach was carried out by Dixon et al., 2023, who reported that LRS accurately detected all the CNVs (from single exons to whole genes, including a tandem duplication) in *BRCA1*, *BRCA2*, *CHEK2* and *PALB2* previously identified in nineteen patients, and defined the precise breakpoints of SVs in *BRCA1* and *CHEK2*, revealing unforeseen allelic heterogeneity and demonstrating the potential for LRS as a powerful genetic testing assay in the hereditary cancer setting. Further confirmations of its diagnostic value are extended to very rare cancer predisposition syndromes. In two siblings both neonatally diagnosed with atypical teratoid rhabdoid tumor, Sabatella et al. applied LRS in combination with optical genome mapping and identified an insertion of a −2.8 kb retrotransposon element within the intron 2 of *SMARCB1*, disrupting its correct splicing and present in mosaic state in the mother (Sabatella et al., 2021).

Also targeted approaches revealed their effectiveness and clinical utility in detecting SVs affecting CSGs. Filser et al. were able to classify a germline duplication event of exons 18–20 of *BRCA1* as pathogenic using adaptive sampling (Filser et al., 2023), while Yamaguchi et al. applied a hybridization-based enrichment to the LRS analysis of mismatch-repair genes responsible for Lynch syndrome, precisely mapping SV breakpoints and proposing their method as a possible substitute for MLPA assays (Yamaguchi et al., 2021).

An additional promising application of LRS is the resolution of cxSV configuration. The study by Sanchis-Juan et al., 2018 provides an example of how LRS can be used to elucidate the structure of a duplication-inversion-duplication in the *CDKL5* gene with breakpoints mapping in highly repeated regions inaccessible by SRS.

Rare cxSVs in the *DMD* gene contribute to the pathogenesis of dystrophinopathies but escape diagnosis by routine diagnostic testing (MLPA and targeted SRS). Gross rearrangements mediated by transposable elements could be missed also by targeted LRS if read length is insufficient to cover the SVs and their breakpoints. Conversely, whole-genome LRS was shown to correctly identify and characterize large-scale cxSVs, contributing to the diagnosis of unsolved cases when implemented as the fourth analysis in a stepwise genetic testing strategy for patients with dystrophinopathies (Xie et al., 2020; Xie et al., 2021). The alpha- and beta-globin genes map in an additional disease-associated locus involved in rare and complex rearrangements that are still underdiagnosed but can be detected by LRS, together with rare SNVs, increasing the diagnostic yield by approximately 7% compared to routine detection methods (variant-tailored Gap-PCR and Sanger sequencing) (Long et al., 2022; Xu et al., 2023; Zhuang et al., 2023).

Thanks to LRS, combined to additional genomic analyses in a multidimensional approach, Scharf et al., 2022 diagnosed a patient with an attenuated familial adenomatous polyposis who remained unsolved by Sanger sequencing and SRS multigene panel for years. They found a complex 3.9 Mb rearrangement involving 14 fragments from chromosome 5q22.1q22.3, which likely originated from chromothripsis events and separated the *APC* promoter 1B from the coding open reading frame, leading to allele-specific downregulation of *APC* mRNA.

All these studies revealed that, although underestimated because still unexplored in diagnostics, cryptic SVs could represent relevant pathogenic events in Mendelian disorders and efficient methods for their detection, such as LRS, should be rapidly translated from the research to the clinical setting. Routine laboratory activities could also benefit from more rapid and less laborious wet lab protocols, remarkably reducing sample processing times compared to conventional techniques. Additionally, the possibility of real-time analysis might significantly decrease the time to diagnosis, with a reported less-than-1-h timeframe for aneuploidies (Magini et al., 2022).

## 3 Tandem repeat-related diseases

Tandem repeats (TRs) are stretches of repeated nucleotide blocks, variable in size (from few to thousands of bases) and highly homologous, thus prone to rearrangements with length-dependent instability. Disorders mainly affecting the central nervous system manifest when the number of repeats exceeds a gene-specific threshold. At least 69 TR-related diseases are known and most of the expanded TRs map within both coding and non-coding regions, typically untranslated regions (UTR) and intronic sequences. Expansions affecting coding TRs generally generate proteins with long polyQ or polyA chains that accumulate within cells or lose their functions. Non-coding expansions have different pathogenic mechanisms, according to their location and length, including gene silencing through methylation for those mapping at the 5′-UTRs (Depienne and Mandel, 2021; Gall-Duncan et al., 2022). Technological limitations negatively affected both their discovery, which accelerated only in recent years, and their diagnosis.

### 3.1 Current diagnostic analyses for expanded TR detection

Three features of these repeated regions make their analysis particularly problematic, especially through PCR-based methods: size, homology, and high GC density.

**FIGURE 1:**
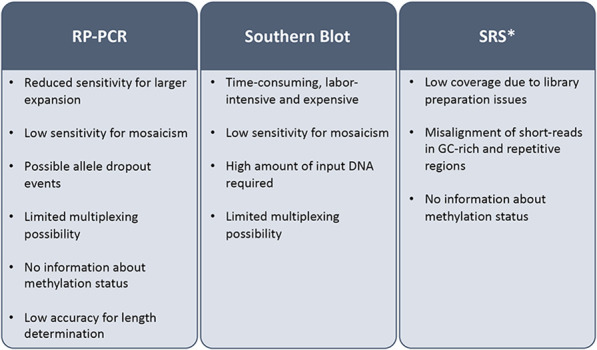
Limitations of current diagnostic tests for tandem-repeat-related diseases. SRS: short read sequencing; RT-PCR: repeat primed-polymerase chain reaction; *its clinical application has been evaluated but technical issues prevented its transition to the diagnostic routine.

Hybridization and correct mapping of PCR primers to target regions are hampered by homologous and GC-rich sequences and polymerase accuracy decreases with long amplicons. For these reasons, specific amplification strategies have been designed (i.e., repeat-primed PCR [RP-PCR]), combined with PCR-free hybridization approaches, such as Southern blot which is still the gold standard for sizing larger expansion. However, these techniques are slow, labor-intensive, not suited to the parallel analysis of multiple loci in a single assay and cannot accurately determine the number of repeats or the structure of the repeated region within very long alleles, which have usually clinical implications, affecting age of onset and disease severity and progression (Cumming et al., 2018; Chintalaphani et al., 2021). Moreover, TR instability may result in somatic mosaicism further challenging consensus size determination and structure definition (Figure 1). The diagnostic yield of these tests ranges from 10% to 30% and their efficacy is impaired for longer (>300 repeats) and complex TRs (Read et al., 2023).

The advent of SRS and the implementation of advanced bioinformatic tools improved the detection and the discovery of repeat expansions (Bahlo et al., 2018), but, although their clinical utility has been demonstrated (Ibañez et al., 2022), the low coverage due to library preparation issues and misalignment of short-reads related to the GC-rich and repetitive nature of these regions, prevented their use as routine diagnostic test (Chintalaphani et al., 2021).

### 3.2 Clinical application of LRS to TR identification

LRS contributed to the discovery of novel disease-associated TRs (Gall-Duncan et al., 2022) and appears as a promising diagnostic method because its whole-genome pre-sequencing protocol avoids PCR-based amplification steps and long reads, sizing over 10 kb, can entirely cover most known pathogenic expanded repeats reaching unique flanking regions, giving specificity to sequence alignment (Mitsuhashi and Matsumoto, 2020). Furthermore, the methylation status of loci can be simultaneously determined, providing an additional relevant disease marker for some conditions.

Although whole-genome LRS is feasible and has been helpful in the discovery of new disease-causing expansions (Corbett et al., 2019; Chintalaphani et al., 2021), target approaches will probably represent more cost-effective strategies to be applied for diagnostic purposes.

Liang et al., 2022 developed and evaluated a long-range PCR and LRS-based assay to analyze the *FMR1* locus on clinical samples. Compared to conventional techniques, target LRS showed higher sensitivity for mosaic expansions, accurate determination of the CGG repeat number of any length, and of AGG interruptions, even in mosaic alleles. This information is extremely important to assess the risk of recurrence and guide reproductive choices. Additionally, the novel approach was able to simultaneously identify intragenic SNVs and repeat-flanking microdeletions potentially disrupting primer annealing and leading to false or negative results through RP-PCR. The turnaround time of the target LRS assay was 8 days and the cost per sample ranged from 40 to 80 $, according to the number of pooled samples. The possibility to combine and sequence together different target assays will further improve the cost-benefit ratio.

Although generally getting a small amount of target DNA, amplification-free enrichment methods, amenable to multiplexing, have been designed by using clustered regularly interspaced short palindromic repeat (CRISPR)-Cas9, avoiding PCR biases, such as preferential amplification of shorter alleles, and issues with repetitive and GC-rich regions.

The combination of CRISPR-Cas9 enrichment and LRS allowed to exclude the presence of interruptions in very long expanded *TCF4* alleles as a penetrance reduction mechanism for Fuchs endothelial corneal dystrophy, at least in some individuals. In addition, it detected novel sequence variants flanking the expansions with a potential role in sizing borderline repeats and confirmed that the heterogeneity in repeat length, previously observed in leukocytes through conventional methods, has a likely biological origin and is not due to PCR artifacts (Wieben et al., 2019). Unamplified DNA enriched for the *DMPK* gene through the CRISPR-Cas9 approach was sequenced by LRS in four patients with myotonic dystrophy type 1 (DM1), revealing more interruptions than those detected by RP-PCR and providing sensitive expanded allele-specific characterization of methylation levels, proposed as more reliable markers of the severe congenital form compared to repeat length. Assessing the length of individual alleles, LRS might also improve the estimation of the progenitor allele length that has been suggested to have a stronger correlation with disease severity and age of onset compared to the modal repeat length of the heterogeneous population of alleles generated by somatic instability (Rasmussen et al., 2022). Compared to amplicon-based enrichment, successfully used by Mangin et al., 2021, the CRISPR-Cas9 approach improved the measurements of repeat length and somatic mosaicism in DM1 patients and the detection of interruptions even within very long expanded alleles. These three clinically relevant parameters (repeat length, somatic mosaicism and interruptions) could be simultaneously analyzed in less than 1 month (Tsai et al., 2022). In addition to the identification of the number of very large repeated units (3.3 kb/unit) in patients with facioscapulohumeral muscular dystrophy, LRS of CRISPR-Cas9-enriched *DUX4* gene detected an atypical pathogenic deletion and provided a methylation profile of the entire D4Z4 locus at nucleotide-level resolution, revealing hypomethylation of the causative contracted allele (Hiramuki et al., 2022). An increased diagnostic yield was obtained by the application of the CRISPR-Cas9 enrichment-LRS approach to a cohort of patients with benign adult familial myoclonic epilepsy, resolving two cases with ambiguous results from RP-PCR/Southern blot on *SAMD12* repeats. Furthermore, whole-genome LRS revealed infrequent, disease-associated repeat expansions in *SAMD12*-negative patients (Mizuguchi et al., 2021). The development of an alignment-independent analysis pipeline simplified the definition of *HTT* repeat sequence in Huntington patients and provided a useful method for the investigation of TRs with variable or unknown length in the reference genome. Although multiplexing with a consequent cost reduction is possible, demanding laboratory protocols, the relevant Cas9-derived off-target issue and the huge input DNA required represent important limitations for the implementation of the CRISPR-Cas9-based LRS in clinical routine (Höijer et al., 2018).

The bioinformatic selection of target regions during the sequencing process through adaptive sampling tools avoids wet-lab-based enrichment procedures and experimental optimizations, reducing DNA amount requirements, costs and analysis times, and allows the simultaneous investigation of hundreds of loci, with easy customization (Chintalaphani et al., 2021).

Stevanovski et al., 2022 tested a custom bioinformatically enriched panel of 37 TR loci associated to neurological or neuromuscular diseases, showing accurate haplotype-resolved sizing, sequence determination and DNA methylation profiling of neuropathogenic short TRs. They also demonstrated the utility of LRS for genotyping very large and complex TRs, with different motif conformations, discriminating between known disease-causing and non-clinically relevant alleles. Fifty-nine disease-associated TR loci have been simultaneously tested through the adaptive sampling method by Miyatake et al. (2022a), that showed higher speed, accuracy and comprehensiveness compared to conventional diagnostic methods, providing suggestions to improve coverage depth and reduce costs.

Additional proof-of-principle studies, small cohort analyses or case reports showed feasibility of LRS application to the characterization of TRs associated to several diseases, including amyotrophic lateral sclerosis and frontotemporal dementia (Ebbert et al., 2018), familial cortical myoclonic tremor with epilepsy (Zeng et al., 2019), neuronal intranuclear inclusion disease (Deng et al., 2019), CANVAS syndrome (Miyatake et al., 2022b), autosomal dominant tubulointerstitial kidney disease (Okada et al., 2022) and synpolydactily (Melas et al., 2022).

The clinical application of an LRS-based comprehensive analysis of expanded TRs, accurately defining repeat length and structure, interruptions, somatic levels and methylation in a single test will improve turnaround times, genotype-phenotype correlations and genetic counseling. The relevance of accurate diagnosis is even more evident in light of the emerging target therapies for some expansion-related diseases, where LRS could be also applied as an effective method to define *in vivo* editing outcomes (Simpson et al., 2023).

However, improvements are needed to make LRS the first-tier test for the diagnosis of TR-related diseases in the future. Despite recent efforts (Mousavi et al., 2019; Liu et al., 2020; Mitsuhashi et al., 2021; Liu et al., 2022b), current population catalogs of repeats are still incomplete, resulting in a hard discrimination between pathogenic and benign repeated tracts (Mousavi et al., 2019; Depienne and Mandel, 2021). Discordant results are generated by different bioinformatic tools, needing more accurate benchmarking (Chintalaphani et al., 2021). Finally, the development of advanced strategies and algorithms is required to improve read depth and base-calling accuracy as high sequencing error rate, low coverage and significant off-target issues observed with some approaches (i.e., CRISPR-Cas9) could impair clinical applications (Ebbert et al., 2018; Tsai et al., 2022).

## 4 Single nucleotide variants

After several decades of Sanger sequencing dominance in the diagnostic field, SRS has revolutionized the detection of SNVs allowing the parallel analysis of many samples and genes. The accuracy and the clinical application of SRS techniques have laid the groundwork for reaching genetic diagnosis in a large proportion of monogenic disorders with a single analysis. However, some pathogenic variants remain undetected, especially if located within difficult-to-access genomic regions. Introducing LRS into clinical practice can overcome these limitations and increase diagnostic yields. Although LRS accuracy in detecting SNVs is not yet comparable to that of the state-of-the-art SRS techniques, recent technological advancements could fill this gap and further foster its diagnostic application, that can help clarify undiagnosed cases and elucidate findings with uncertain clinical significance (Lin et al., 2021; Ni et al., 2023).

### 4.1 Pseudogenes

As previously mentioned, one of the most important limitations of SRS is the inability to read repetitive or duplicated regions of the genome. Mapping highly homologous sequences is complicated and the incorrect alignment of reads can lead to either false negative or positive results. The analysis of these loci usually requires more than one technique to obtain a reliable result, and the process is time-consuming and highly dependent on the expertise of the center where the analysis is performed. Conversely, LRS can access and accurately map these genomic regions and successfully detect variants in genes with a high degree of homology with one or more pseudogenes, providing an efficient single-test tool.

The classic example of a difficult-to-test gene is *PKD1*, which is characterized by high GC-content, high homology with six pseudogenes and a heterogeneous mutational spectrum. The SRS analysis of this gene requires a long-range PCR-based amplification of coding exons through specific primers anchored to mismatched sequences between *PKD1* and pseudogenes. To further increase mapping accuracy, reads are aligned against a modified reference genome masking nucleotides outside the *PKD1* locus. Compared to Sanger sequencing, this strategy showed 100% sensitivity, but 63% specificity and 92% accuracy, with false positive calls in GC-rich regions and homopolymers, and allele dropout issues (Mantovani et al., 2020). MLPA should be applied on patients with wild type *PKD1* sequence to identify possible pathogenic CNVs.

Borràs et al. applied LRS on pooled long-range-PCR amplicons to screen for *PKD1* mutations in 19 patients with autosomal dominant polycystic kidney disease, enabling to distinguish between *PKD1* and its pseudogenes and reducing the interference of homologous sequences, high GC-content or repetitive elements (Borràs et al., 2017). The technique allowed the identification of 20 out of 21 pathogenic variants including substitutions, single nucleotide deletions, large deletions, one deletion-insertion and three out of four insertions or duplications, providing a correct diagnosis in 18/19 patients. One pathogenic insertion was not detected by LRS despite high read depth, most likely due to noise in homopolymer sequencing. The authors suggest that LRS could be a possible alternative technique to those currently used to enable *PKD1* screening with a single diagnostic test, although further studies on larger cohorts are required before implementing this strategy into the clinic.

Several authors have applied LRS to solve sequencing difficulties related to *CYP21A2*. Biallelic mutations in *CYP21A2* are responsible for autosomal recessive congenital adrenal hyperplasia. This gene has a high degree of homology with its nonfunctional pseudogene, *CYP21A1P*, which is located in tandem with the functional gene. Together with the adjacent genes and relative pseudogenes, they form a genetic module called RCCX (*RP1-C4A-CYP21A1P-TNXA-RP2-C4B-CYP21A2-TNXB*), where the high frequency of misalignments leads to gene conversions, structural rearrangements and formation of chimeric genes. Currently, diagnostic testing mostly relies on MLPA and Sanger sequencing, both with relevant limitations preventing an accurate analysis of this complex locus and often leading to ambiguous results. Long-range locus-specific PCR combined with LRS successfully identified SNVs, chimeric genes and complex rearrangements, providing a comprehensive analysis of *CYP21A2* mutations with 100% sensitivity and specificity when compared to traditional methods (Liu Y. et al., 2022; Tantirukdham et al., 2022; Li et al., 2023). Moreover, LRS allowed the direct detection of copy number and cis-trans configuration of *CYP21A2* and *CYP21A1P* without the need to test other family members. However, as long-range-PCR is needed, there is a residual risk of allele dropout during amplification. All things considered, LRS was proved to be a promising, cost and time effective technique for carrier screening and diagnosis of congenital adrenal hyperplasia.

Further studies have shown that LRS is suitable for the analysis of genes with important epidemiological significance and whose sequencing is complicated by the presence of pseudogenes, such as for spinal muscular atrophy (SMA). SMA diagnosis deserves particular attention because of recent great progress in the implementation of new therapies with outcomes critically related to early administration and consequently to the availability of efficient newborn screening tests for the rapid identification of affected children (Schorling et al., 2020). SMA is an autosomal recessive disease caused by biallelic mutations in *SMN1*, that has a high degree of homology with neighboring *SMN2* gene. The number of copies of *SMN2* is variable in the population and is inversely correlated with clinical severity (Kolb and Kissel, 2015). Most patients (95%) carry homozygous deletion of *SMN1*; less frequently, the mutation is represented by a SNV/indel (Bai et al., 2024). Carrier screening in parents or in healthy at-risk population is needed to assess recurrence risk in offspring. Current methods for SMA diagnosis involve a multiple-step, time-consuming process, making use of *SMN1* dosage tests (MLPA or quantitative PCR for copy number detection), followed by *SMN1* sequencing when a heterozygous deletion is identified (Bai et al., 2024). Moreover, conventional dosage tests for *SMN1* fail to identify the *SMN1* 2 + 0 genotype, a carrier status characterized by two copies of *SMN1* on the same chromosome. To overcome these limitations, LRS is being tested as a single-step method for SMA diagnosis and carrier screening. It correctly identified CNVs, SNVs/indels and large deletions in *SMN1*, *SMN2* copy number and modifying SNVs (Bai et al., 2024) and detected *SMN1* 2 + 0 carriers (Chen et al., 2023). Additional large-scale studies are needed to safely implement the technique as a single-test screening for this condition, but the results to date are promising.

### 4.2 Haplotype phasing

In autosomal recessive diseases, haplotype phasing of variants is essential to determine the genetic diagnosis in compound heterozygote cases. According to the guidelines of the American College of Medical Genetics (ACMG), the detection of a genetic variant in *trans* with a second pathogenic mutation is moderate evidence of pathogenicity (PM3) and this criterion can help redefine variants of unknown significance (VUS) as likely pathogenic/pathogenic (Richards et al., 2015). However, variant phasing by short-read or Sanger sequencing requires the analysis of patients’ parents or other family members and can be complicated by their unavailability. This situation is unfortunately frequently encountered in clinical practice. Recently, Gupta et al. reported on a proband affected by visual and hearing impairment in whom genetic testing revealed two variants in the *USH2A* gene, a pathogenic variant and a VUS. Parental testing was not possible, but a single LRS read covering the genomic positions of both variants demonstrated their localization in *trans*. This allowed the reclassification of the VUS as likely pathogenic and confirmed the diagnosis of Usher syndrome in the proband (Gupta et al., 2023).

Diagnostic uncertainty in autosomal recessive diseases also remains when parental samples are available, but one of the two variants occurred *de novo* in the proband. Again, LRS can resolve the phasing of variants and define their clinical relevance (Zhao et al., 2021).

The identification of the parental allele affected by a *de novo* variant, can have important implications not only for autosomal recessive genes. Indeed, in imprinting disorders, the parental origin of the mutated allele determines its clinical significance. For instance, Watson and colleagues reported on a proband with a clinical diagnosis of Angelman syndrome (AS) and a potentially pathogenic heterozygous variant in *UBE3A*. To confirm the diagnosis, the maternal origin of the mutation should have been verified, but DNA analysis of both parents was negative for the specific variant. LRS allowed the reconstruction of the parental haplotypes and demonstrated the maternal origin of the mutated allele (Watson et al., 2022). This example illustrates the usefulness of LRS in imprinting disorders besides its ability of detecting methylation alterations (see below).

### 4.3 Lost in SRS

Most SRS assays just study the coding sequence and the canonical splice sites through whole exome sequencing (WES). Besides the identification of missed coding variants in difficult-to-sequence regions, several case reports and small-cohort studies showed how LRS helped the detection of intronic variants located outside of the canonical splice-site that weren’t previously identified by standard genetic techniques (Bruels et al., 2022; Miller et al., 2022; Viering et al., 2023). As a matter of fact, most of these variants would likely be identified with short-read WGS and limitations remain in computational prediction algorithms for deep intronic variant interpretation. Nevertheless, there are advantages in LRS over WGS, such as the ability to simultaneously detect potential SVs and phasing the different variants, in addition to a possible cost-effectiveness when targeted approaches are applied. We suggest that LRS might transiently become a valid second-line approach for the diagnosis of patients with inconclusive findings after standard analyses, such as SRS gene-panels or WES. However, its application as first-tier diagnostic test will have to wait for technological advances solving technical limitations and the development of analysis software easily manageable by biologists without bioinformatic expertise.

## 5 Methylation changes at imprinted genomic regions

Detecting methylation differences can be useful in defining the diagnostic classification of DNA sequence changes, such as in repeat expansion disorders, where LRS can simultaneously detect both DNA sequence alterations and base modifications (Miller et al., 2022). Nevertheless, at present the main diagnostic application of methylation analyses concerns the identification of defects responsible for imprinting disorders. Imprinting disorders are caused by genetic or epigenetic alterations affecting differentially methylated regions that regulate the monoallelic and parent-of-origin-dependent expression of imprinted genes. The underlying molecular defects are heterogeneous (uniparental disomy [UPD], CNVs, SNVs and methylation changes), contribute with different frequencies to the occurrence of the different clinical conditions, and sometimes arise in postzygotic cells. Molecular heterogeneity, mosaicism and the frequent absence of specific clinical manifestations make diagnosis challenging. Identifying the molecular cause is important not only for better clinical management, as for other genetic diseases, but also for surveillance of tumor risk occurring in some imprinting disorders (Beygo et al., 2020).

### 5.1 Current diagnostic analyses for imprinting disorders

Accurate estimates of diagnostic yield for individual imprinting disorders are not available as this is influenced by the stringency level of referral clinical indications resulting in lower diagnostic rates in patients routinely analyzed by genetic laboratories compared to those achieved in clinically well-characterized cohorts (Mackay et al., 2022; Eggermann et al., 2023). The diagnostic workflow of a specific disorder depends on the frequencies of the different molecular mechanisms and on the expertise of laboratories, but generally, for most conditions, such as AS and Prader-Willi (PWS) or Beckwith-Wiedemann (BWS) and Silver Russell syndrome, mainly due to UPD, CNVs or imprinting defects, all leading to changes in the methylation pattern at specific genomic loci, it starts with tests targeting these epigenetic modifications.

Epigenetic modifications are often studied using bisulfite conversion of unmethylated cytosines to uracil, that are eventually converted to thymidines during PCR amplification, while 5-methylated cytosines (5 mC) are protected from conversion. These methods allow genome-wide methylation profiling at a single-base level, although C>T conversion creates mapping difficulties and bisulfite treatment can alter DNA integrity.

Methylation-Sensitive (MS)-MLPA, which simultaneously detects altered methylation and CNVs, has replaced MS bisulfite conversion-dependent methods and is now routinely applied as a first-tier test for clinically suspected imprinting disorders. This technique analyzes native DNA through methylation-sensitive restriction enzyme digestion, but cannot distinguish between UPD and imprinting defects with few exceptions, needing second-line assays to identify the exact underlying defect and thus enabling adequate genetic counseling with accurate estimation of recurrence risks (Beygo et al., 2020; Eggermann et al., 2023). Reduced sensitivity for low-level mosaic alterations and allele dropout are major limitations, with the latter mitigated by using more probes for single loci. Additionally, commercial MS-MLPA kits generally test for single or few chromosomes requiring the sequential application of more kits in patients with phenotypic features compatible with clinically overlapping imprinting disorders and are unsuitable for multi-locus imprinting disturbances. The adoption of a multi-locus imprinting test might be useful to increase diagnostic yield (Mackay et al., 2022). Microsatellite analysis is the gold standard test for UPD involving either whole or partial chromosomes (segmental UPD). Probands and both parents must be tested in parallel to establish the parental origin of all the markers analyzed. The same trio strategy should be applied when using SNP-arrays to be able to detect heterodisomy in addition to isodisomy (Beygo et al., 2020).

### 5.2 Application of LRS to the diagnosis of imprinting disorders

A single assay investigating all the possible molecular pathogenic mechanisms simultaneously will speed up diagnosis, prognosis and therapy, through the rapid definition of (epi)genotype-phenotype correlations. LRS allows the detection of modified or methylated nucleotides (including N6-methyladenine, 5mC and 5-hydroxymethylcytosine) directly in the native genomic DNA during canonical DNA sequencing, without bisulfite treatment. In real-time single molecule sequencing approaches, methylation impacts the kinetics of the polymerase while incorporating fluorescently labeled nucleotides into nucleic acid strands during DNA sequencing: base modifications alter both pulse width and interpulse duration, allowing the detection of epigenetics changes (Flusberg et al., 2010). In nanopore-based technologies, the electric current pattern is different for modified and non-modified bases and methylation state can be predicted with specific tools (Liu et al., 2021).

Few studies tested the clinical application of LRS for the diagnosis of imprinting disorders, probably due to the still high costs of the technology. Long-read genome sequencing has been recently used to analyze two patients with imprinting disorders, identifying a loss of methylation at IC2 locus at 11p15 responsible for BWS in one case and maternal UPD at 15q11 causing PWS in the other case (Dixon et al., 2022). The genome-wide approach also allowed researchers to investigate the additive contribution of possibly pathogenic variants in different genes to the clinical picture of patients. More patients (18) with a clinical diagnosis of AS or PWS were analyzed through LRS and standard clinical testing by Paschal et al., 2023, observing a 100% concordance between methods, without specifying the molecular causes.

As mentioned before, a possible solution to decrease costs is the adoption of a target strategy.

Yamada et al., 2023 demonstrated that targeted LRS through adaptive sampling might be an efficient one-step assay for the diagnosis of AS and PWS and potentially other imprinting disorders. They correctly identified alleles with altered methylation, which is sufficient for diagnosis, and were allowed to discriminate large and very small (imprinting center) deletions, uniparental isodisomy and heterodisomy, which is useful for genetic counseling and clinical management. Costs could be further reduced by including additional clinically relevant regions in the target analysis, as also technically recommended for the adaptive sampling approach that requires relatively large target size.

More data about sensitivity in additional disease-causing differentially methylated regions and for mosaic alterations of methylation are needed to confirm LRS clinical utility for the diagnosis of imprinted disorders.

## 6 Alterations of gene expression: to be included within diagnostic workflows

RNA sequencing (RNAseq) technologies allow the quantitative and qualitative analysis of gene expression in different cells and tissues, enabling the reconstruction of the transcriptome, that is, the set of coding and noncoding transcripts. Among its potential applications, RNAseq can be of use in the diagnosis of rare genetic disorders to identify differentially expressed isoforms and alternative splicing, detect gene fusions and predict the functional impact of genetic variants (especially non-coding variants), increasing the diagnostic yield by up to 15% compared with WES alone (Peymani et al., 2022).

The presence of diverse transcripts for a single genomic locus can raise problems in the assembly process, complicating transcript identification from short reads (de Jong et al., 2017). LRS can overcome these difficulties and simultaneously retrieve phasing information, although there are still few examples of its application in a clinical setting, just as the implementation of RNA analysis in diagnostic workflows is still limited.

Dainis et al., 2019 reported on a patient with severe hypertrophic cardiomyopathy (HCM) in whom a splice-site variant was identified in *MYBPC3*, the most frequently mutated gene in HCM. The variant was predicted to disrupt exon 19 to 20 splice junction. With targeted LRS, the authors identified a novel, highly expressed, alternatively spliced transcript (missing exon 20) in the patient’s cardiomyocytes and, with phasing information, they reconstructed that the alternative isoform was generated by the mutant allele. In this case, RNAseq allowed the characterization of the variant and provided evidence of pathogenicity.

For an effective application of long-read RNAseq in a clinical setting, automated workflows are required. For example, a capture and ultradeep long-read RNA sequencing (CAPLRseq) workflow was developed and validated for the diagnosis of hereditary non-polyposis colorectal cancer/Lynch syndrome that enables the interpretation of variants affecting mRNA structure or expression in 123 hereditary cancer gene transcripts (Schwenk et al., 2023). This method allowed the reclassification of VUS in two patients (one likely benign and one likely pathogenic variant) and confirmed the presence of splicing defects or alterations in the expression of mismatch repair genes in 17 more patients. The variants detected included coding and non-coding SNVs and SVs that resulted in premature termination of transcript, splicing defects, monoallelic loss of expression, insertion of retrotransposons or formation of fusion transcripts.

Despite its usefulness in resolving the clinical role of uncertain variants, the implementation of long read RNAseq in clinical practice is still limited by the high cost, the practical difficulties in obtaining suitable tissues for analysis and the need for more automated workflows.

## 7 Discussion

After SRS, the last huge advancement in the field of genomic and genetic research is represented by the advent of LRS, which proved to be a powerful tool to rapidly analyze genomes, epigenomes and transcriptomes. Its ability to accurately sequence complex genomic regions allowed to sequence the first telomere-to-telomere complete human genome, which earned it the nomination as method of the year 2022. Although LRS is increasingly applied in research, its use in diagnostics is not yet part of routine clinical practice. In this review, we focused on the potential diagnostic applications of the technique, highlighting its advantages and limitations (Table 2).

**TABLE 2:** Advantages and limitations of long read sequencing clinical application for the detection of different genetic/epigenetic anomalies. SRS, short read sequencing; SNV, single nucleotide variant; SV, structural variant; TR: tandem repeat.

	SVs	TR expansion	SNVs	Methylation changes
ADVANTAGES	Accurate definition of breakpoints	Precise determination of the repeat number in individual alleles, regardless the length	Identification and correct mapping of variants in genes highly homologous to pseudogenes	Definition of the underlying pathogenic mechanism
	Mapping of duplicated regions	Improved estimation of the progenitor allele length	Direct haplotype phasing without the need of parental testing	Allele discrimination
	Resolution of complex rearrangements	Accurate identification of interruptions		High sensitivity for mosaic changes
	Identification of SVs involving repetitive regions	High sensitivity for mosaic expansions		
	High sensitivity for mosaic alterations	Parallel detection of methylation changes		
	Identification of missed alterations in the second allele of recessive genes			
	Rapid wet-lab and analysis protocols			
	Possibility of targeted analysis with easy multiplexing and customization (adaptive sampling)			
LIMITATIONS	Reduced accuracy for SNV detection compared to SRS			
	Interpretation difficulties			
	High costs (containable with targeted approaches)			
	Bioinformatic expertise required			

One of the main benefits of LRS, which is clearly reflected in the available literature, is the detection of balanced and unbalanced SVs, including complex rearrangements, with high sensitivity and accuracy, through reliable alignment and precise breakpoint definition. Moreover, for the detection of expanded TRs, LRS outperforms current diagnostic techniques in both accuracy and practicality. Considering also the availability of efficient targeted solutions, we suggest that LRS could become the diagnostic method of choice for these types of genetic alterations in the next few years, providing significant improvements in terms of diagnostic yield and time to diagnosis. In line with these highlighted capabilities, many authors have reported the usefulness of LRS in a diagnostic setting, especially in cases previously unsolved by standard techniques and with a strongly suspected genetic etiology. However, at present LRS performances for SNV detection are not yet comparable to those of SRS, preventing its application as a first-tier diagnostic test. Although technical advancements have led over time to the release of new kits and protocols providing greatly improved sensitivity and accuracy, extensive studies are still needed to ascertain their clinical potential. By accessing difficult-to-sequence regions, an additional feasible diagnostic application, replacing current genetic tests, might be the analysis of disease-associated genes highly homologous to pseudogenes (e.g., *PKD1*, *CYP21A2*, *SMN1*), in which the high coverage achieved through targeted approaches might reduce the impact of sequencing errors and allow the identification of SNVs along with SVs, in a comprehensive one-step assay.

The ability to detect methylation changes gives LRS a huge advantage over previous sequencing techniques, but with a currently limited clinical impact on medical genetics. Indeed, methylation evaluation in genetic laboratories is aimed only at the diagnosis of imprinted disorders. The method mainly used in diagnostic routine, MS-MLPA, is sufficiently sensitive, robust and cost-effective and is based on easy wet lab protocols and very user-friendly analysis software. These features make the replacement of MS-MLPA with LRS highly unlikely at least in the next few years, despite LRS greater sensitivity for mosaic alterations and its ability to screen the whole genome and give complete information regarding the pathogenetic molecular mechanisms that have implications for the clinical management of patients and families. The emerging clinical relevance of altered methylation patterns, representing episignatures of specific genetic disorders (Aref-Eshghi et al., 2019), will probably open a new way for LRS to be useful in the field of medical epigenetics.

The present review focuses on diagnostic tests provided by genetic laboratories for the identification of rare genetic variants with strong effects on constitutional phenotypes or hereditary tumor risk. However, further future expansion of LRS clinical applications may include the dissection of cancer cellular heterogeneity to improve diagnosis and targeted therapies through single-cell genomics and the genotyping of common variants for polygenic risk score (PRS) estimation in complex diseases. Currently, these approaches do not have a clearly assessed clinical relevance and are affected by technical and methodological limitations preventing their implementation within diagnostic workflows, despite intense research and evidence provided on their potential.

Indeed, single-cell analysis, even combined to LRS, is mainly used to investigate the biological complexity of cell types and tissues, especially in cancer, with studies primarily aimed at developing efficient and accurate analytical methods (Hård et al., 2023; Shiau et al., 2023; Wu and Schmitz, 2023; You et al., 2023). At present, published work in single-cell genomics remains firmly in the research space and exploration of clinical relevance, especially in the fields of oncology, immunology, and hematology, is still at an early stage (Kim N. et al., 2021; Lim et al., 2023).

Common variants can be combined to define a PRS, estimating the individual susceptibility to complex diseases. Since the clinical utility of this score has not been fully established, due to disease risk prediction challenges, low power in the general population and weak evidence in non-European ancestry, the clinical interest is limited to use PRS in specific cohorts with high prior probability of disease for risk stratification and identification of individuals to be addressed to screening programs, lifestyle modifications, or preventive treatment, when available and appropriate (Lewis and Vassos, 2020). Microarrays have generally been used to detect known common variants associated to diseases and clinically tested to infer PRS (Hao et al., 2022), and whole genome sequencing, extending the analysis to rare variants, may become an appealing alternative, especially through the cost-effective low-pass sequencing strategy (Kim S. et al., 2021; Bagger et al., 2024). An interesting saving-money approach is the analysis of discarded off-target reads from SRS gene panels to reconstruct genome-wide germline genotypes (Gusev et al., 2021). In these perspectives also LRS may give its contribution and a very recent study about targeted LRS on patients with suspected hereditary cancer shows that off-target reads can be effectively used to accurately genotype common SNPs across the entire genome, enabling PRS calculation (Nakamura et al., 2024).

Despite strong evidence of great diagnostic potential, for many laboratories the access to LRS is still complicated by the high cost of both instrumentation and reagents.

However, it should be considered that the high economic commitment for LRS analysis is mitigated by the feasibility of performing different levels of analysis (e.g., SNVs, SVs, methylation) in the same experiment, avoiding the use of multiple and sequential unnecessary tests. Moreover, in cases where a whole-genome approach is not necessary, the cost can be lowered by targeted strategies.

Besides costs, the widespread adoption of LRS in diagnostic laboratories could be slowed down by the high level of bioinformatic expertise required for data analysis, generally available only in big diagnostic centers, and by interpretation difficulties of the huge number and variety of detectable variants, especially due to the incomplete knowledge about genomic regions that have been inaccessible so far.

**FIGURE 2:**
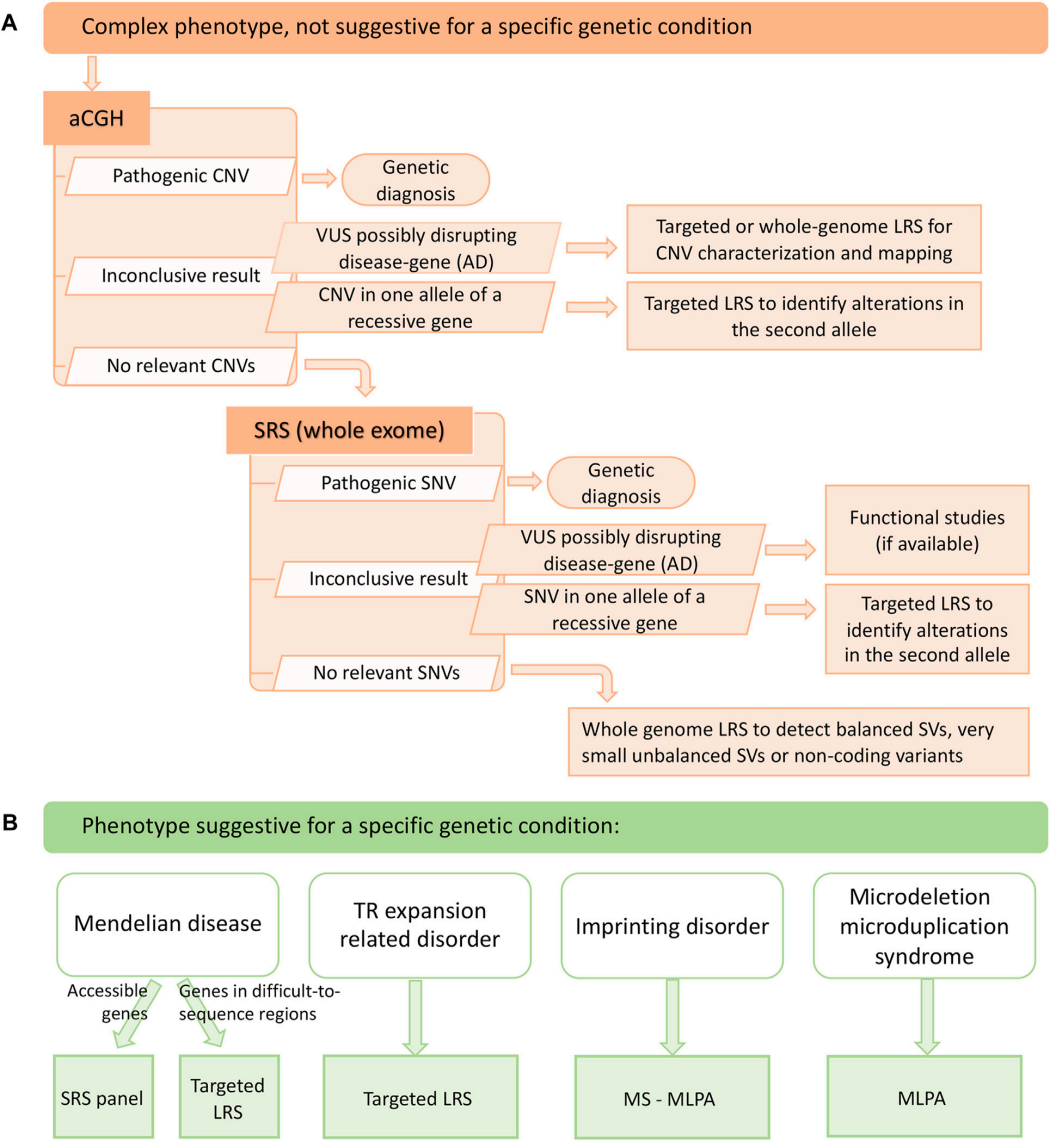
Suggested diagnostic workflows that include long read sequencing. **(A)** diagnostic workflow to be adopted in case the patient presents with a complex phenotype, not suggestive for a specific genetic condition. Long read sequencing can help define copy number variants of uncertain significance, a targeted approach can be useful to study the second allele of a recessive gene when a monoallelic variant is identified and, finally, a whole genome approach can be an additional diagnostic tool in case neither single nucleotide variants nor copy number variants are detected with other techniques. **(B)** diagnostic workflow to be adopted in case the patient presents with clinical features that are suggestive for a specific genetic condition. Targeted long read sequencing can help diagnose mendelian diseases involving difficult-to-sequence genes and tandem repeat-related disorders. aCGH: array-comparative genomic hybridization; AD: autosomal dominant; CNV: copy number variant; LRS: long read sequencing; MLPA: multiplex ligation-dependent probe amplification; MS-MLPA: methylation-specific multiplex ligation-dependent probe amplification; SRS: short read sequencing; SNV: single nucleotide variant; SV: structural variant; TR: tandem repeat.

Considering advantages and limitations, we propose two diagnostic workflows for suspected genetic conditions with or without specific clinical hypotheses, where LRS could be implemented as a second-level test for completing results from previous investigations and as a first-tier test for TR-related disorders and diseases associated to genes in repetitive or GC-rich regions, especially those known to be frequently affected by SVs. Its remarkable ability to identify and characterize SVs should also promote the use of LRS in cases where aCGH and SRS are negative (Figure 2).

Improvements in SNV detection and studies confirming their clinical accuracy are particularly awaited for the possibility of fully characterizing the genome in a single assay. Efforts are being made to overcome the limitations of LRS and make it more widely available.
